# Risk-Stratified Screening: A Simulation Study of Scheduling Templates on Daily Mammography Recalls

**DOI:** 10.1016/j.jacr.2024.12.010

**Published:** 2025-03

**Authors:** Yannan Lin, Anne C. Hoyt, Vladimir G. Manuel, Moira Inkelas, Mehmet Ulvi Saygi Ayvaci, Mehmet Eren Ahsen, William Hsu

**Affiliations:** a Medical & Imaging Informatics, Department of Radiological Sciences, David Geffen School of Medicine at the University of California, Los Angeles (UCLA), Los Angeles, California.; b Department of Radiological Sciences, David Geffen School of Medicine at UCLA, Los Angeles, California.; c Medical Director, Barbara Kort Women’s Imaging Center, Santa Monica, California.; d Medical Director, Santa Monica-UCLA Integrated Breast Care Clinic, Santa Monica, California.; e Co-Medical Director of Breast Imaging, UCLA Health.; f Department of Family Medicine, David Geffen School of Medicine at UCLA, Los Angeles, California.; g UCLA Clinical and Translational Science Institute, Los Angeles, California.; h Department of Health Policy and Management, UCLA Fielding School of Public Health, Los Angeles, California.; i Co-Lead, Population Health Program, UCLA Clinical and Translational Science Institute, Los Angeles, Califonia.; j Co-Lead, UCLA TL1 Translational Science Training Program, Los Angeles, California.; k Area Coordinator, Healthcare Management, Naveen Jindal School of Management, The University of Texas at Dallas, Richardson, Texas.; l Department of Business Administration, Gies College of Business, University of Illinois at Urbana-Champaign, Champaign, Illinois.; m Department of Biomedical and Translational Sciences, Carle Illinois School of Medicine, University of Illinois at Urbana-Champaign, Urbana, Illinois.; n Medical & Imaging Informatics, Department of Radiological Sciences, David Geffen School of Medicine at UCLA, Los Angeles, California; Deputy Editor, *Radiology: Artificial Intelligence*.; o Department of Bioengineering, University of California, Los Angeles, Los Angeles, California.

**Keywords:** appointment scheduling, artificial intelligence (AI), breast cancer screening, clinical workflow, risk-stratified screening

## Abstract

**Introduction:**

Risk-stratified screening (RSS) scheduling may facilitate more effective use of same-day diagnostic testing for potentially abnormal mammograms, thereby reducing the need for follow-up appointments (“recall”). Our simulation study assessed the potential impact of RSS scheduling on patients recommended for same-day diagnostics.

**Methods:**

We used a discrete event simulation to model workflow at a high-volume breast imaging center, incorporating artificial intelligence (AI)-triaged same-day diagnostic workups after screening mammograms. The RSS design sequences patients in the daily screening schedule using cancer risk categories developed from Tyrer-Cuzick and deep learning model scores. We compared recall variance, required hours of operation to accommodate all patients, and patient wait times using traditional (random) and RSS schedules.

**Results:**

The baseline simulation included 60 daily patients, with an average of 42% receiving screening mammograms and 11% (about three patients) being recommended for diagnostic workups. Compared with traditional scheduling, RSS scheduling reduces recall variance by up to 30% (1.98 versus 2.82, *P* < .05). With same-day diagnostics, RSS scheduling had a modest impact, increasing the number of patients served within normal operating hours by up to 1.3% (55.4 versus 54.7, *P* < .05), decreasing necessary operational hours by 12 min (10.3 versus 10.5 hours, *P* < .05), and increasing patient waiting times by an average of 2.4 min (0.24 versus 0.20 hours, *P* < .05).

**Conclusion:**

Our simulation study suggests that RSS scheduling could reduce recall variance. This approach might enable same-day diagnostics using AI triage by accommodating patients within normal operating hours.

## INTRODUCTION

Real-time interpretation of mammograms and same-day diagnostic imaging after potentially abnormal mammograms shorten the time between screening and definitive results and eliminate the need for follow-up scheduling [1,2]. This approach has been implemented in several health systems in the United States but is uncommon [3–5]. Most breast imaging center radiologists interpret screening mammograms in batches after patients have left the clinic. Patients requiring further imaging are notified and scheduled for diagnostic imaging at a later date. Artificial intelligence (AI) tools for mammography could provide immediate signals of which patients to invite for same-day diagnostics, thereby enabling same-day diagnostics. However, accommodating same-day diagnostics is an operational challenge for clinics and could create bottlenecks given fixed technologists, radiologists, and physical resources. Excessive variation in the number of recalled patients and the diagnostic requirements of those receiving same-day diagnostics is a barrier to adoption.

In earlier studies, we simulated incorporating AI-triaged same-day diagnostics with an immediate review by a radiologist in a high-volume breast imaging center [6,7]. Using discrete event simulation, a technique that models the operation of a system as a discrete series of events in time, aiming to evaluate, predict, and optimize an existing or proposed system [8], we examined the impact of the AI-triaged same-day diagnostic workup on clinical operations (eg, operating hours) and patient experience (eg, waiting times). The average percentage of patients recalled from screening mammography was 12%, ranging from 0 to 14 per day. Such variation can hinder the operational feasibility of same-day diagnostics.

We hypothesize that scheduling templates with appointments based on patient risk for developing breast cancer could serve as a surrogate to predict the likelihood of recall from screening mammography and reduce daily variation. We examined how a risk-stratified screening (RSS) schedule affects the daily number of recalls and patient care requirements (eg, the clinic’s ability to accommodate all scheduled screening mammograms and same-day diagnostics within normal operating hours) relative to a conventional nonrisk-stratified scheduling system. The RSS integrates the Tyrer-Cuzick risk assessments [9] with outputs from a deep learning-based AI risk prediction model [10] and known predictors of recall [1].

## METHODS

Institutional review board approval was obtained at the University of California, Los Angeles. We followed four key steps, planning, modeling, verification, and validation (refer to the Results section), and analysis, to develop and validate the simulation models [11]. We built from previous models [6,7] to compare RSS and non-RSS templates. In this study, the term “recall” refers to the diagnostic care after a potentially abnormal screening mammogram, which may involve either same-day diagnostics or follow-up diagnostic workups at a later date.

### Setting

Our simulations used data from the highest-volume breast imaging center within our health system, which conducts approximately 20% of all breast imaging examinations. This site offers all types of breast examinations and procedures, including screening mammography and ultrasound, breast MRI, diagnostic breast imaging, and multimodality image-guided biopsies. Current practice and patient volume served as the baseline workflow. A team expert in imaging, informatics, and implementation science developed a hypothetical workflow incorporating AI interpretation of screening mammograms, immediate radiologist review of flagged results, and same-day diagnostic imaging.

### Modeling

**Fig. 1.**
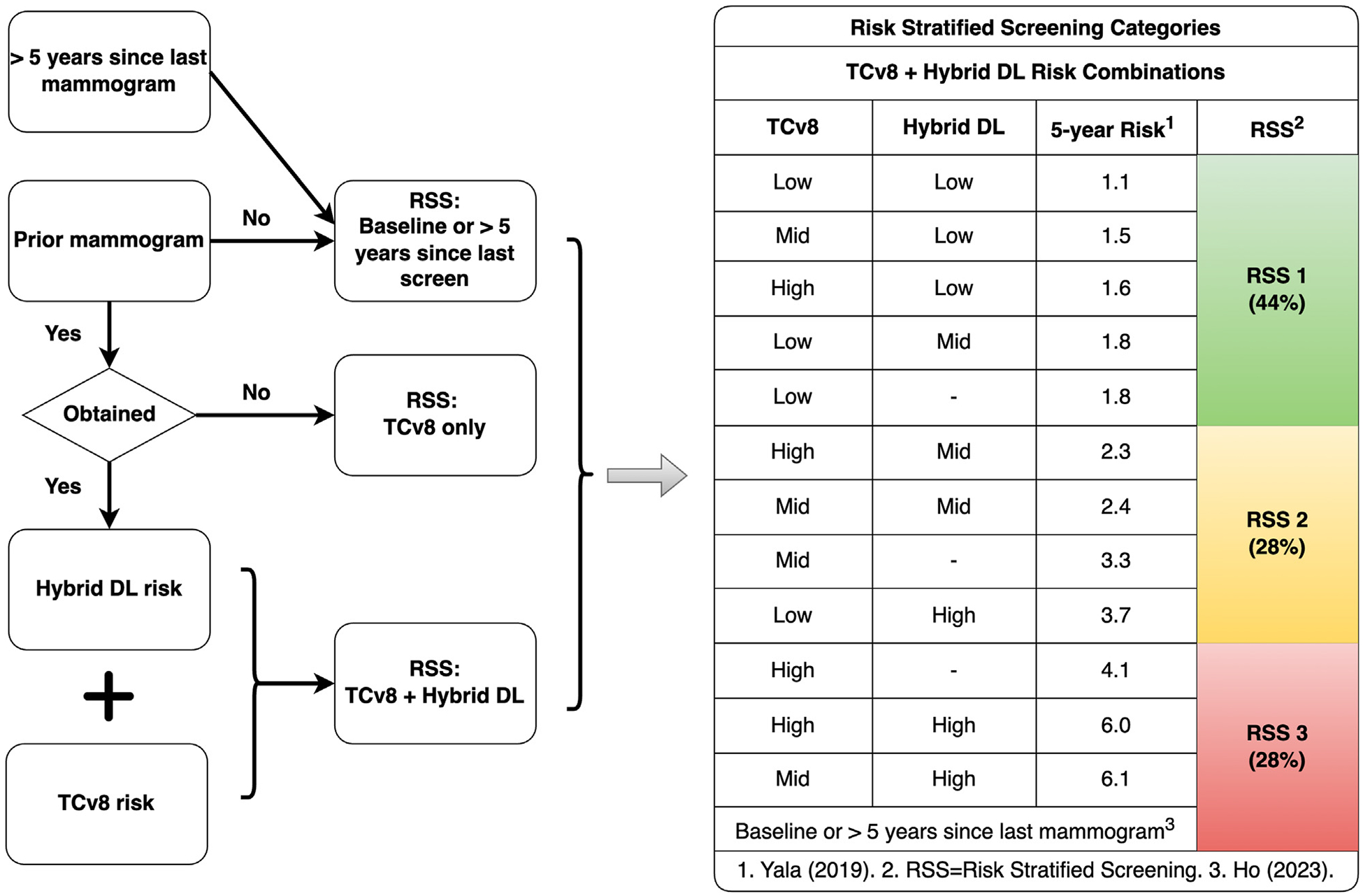
RSS scheduling flow and categories. The RSS categories were derived with data from the Tyrer-Cuzick risk assessment [9], output from a deep learning-based AI risk prediction model [10], and known predictors of recall [1]. DL = deep learning model; RSS = risk-stratified screening; TCv8 = Tyrer-Cuzick version 8.

**Fig. 2.**
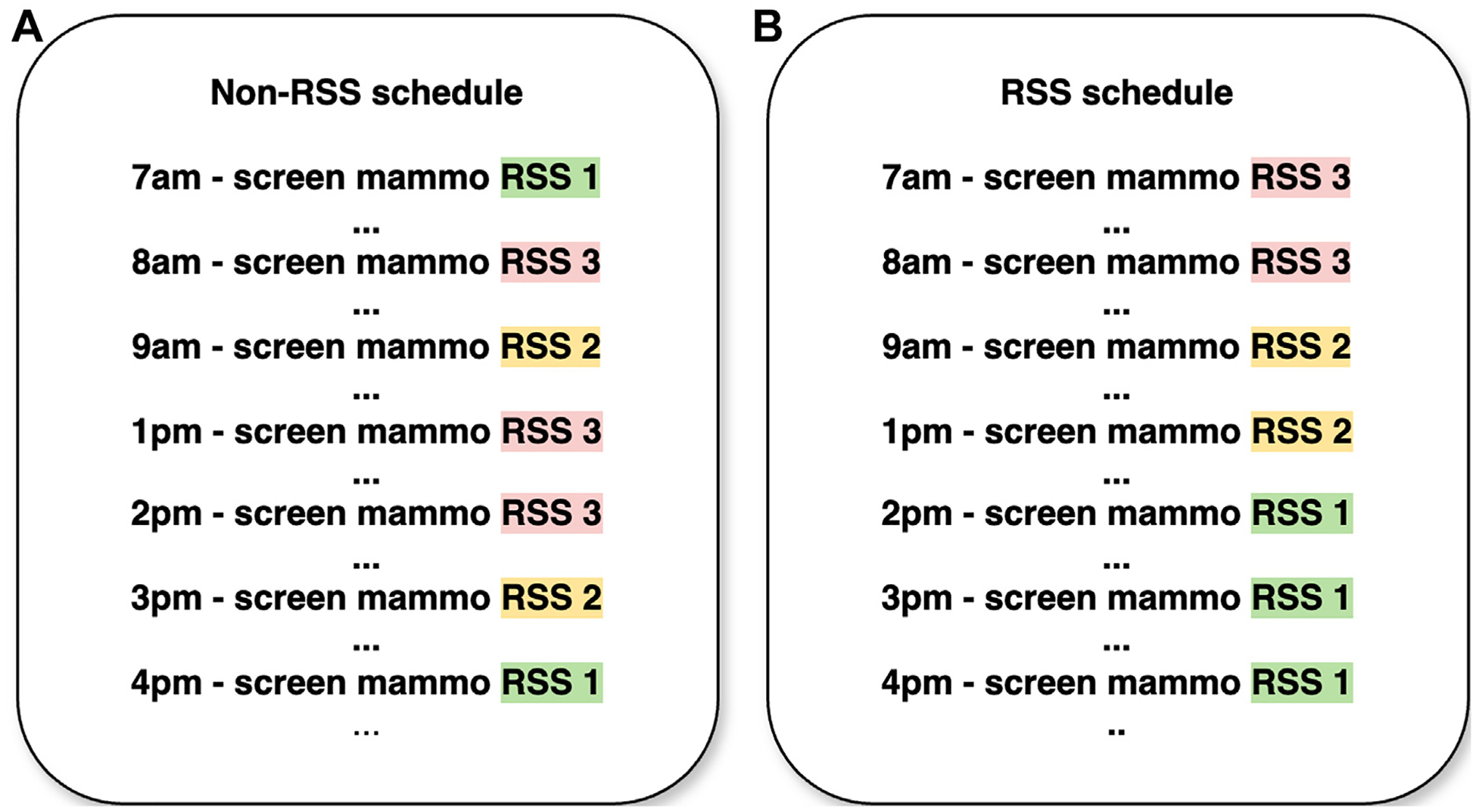
Examples of scheduling templates with or without RSS. (a) Non-RSS: All appointments are equivalent and open to any patient with any risk of developing breast cancer. (b) RSS: Appointments are stratified by patient risk, with higher-risk appointments scheduled earlier in the day than lower-risk patients. The patient may only book an appointment that matches their risk of developing breast cancer. mammo = mammogram; RSS = risk-stratified screening.

Two scheduling templates were examined: non-RSS (baseline scheduling) and RSS. Figure 1 illustrates RSS categories defined by (1) a 5-year Tyrer-Cuzick risk score [9] and (2) a hybrid deep learning model for predicting breast cancer risk (see e-only Supplemental Methods) [10]. Patients without a baseline screening mammogram or one in the past 5 years are placed in the elevated recall risk category [1]. In the non-RSS template (Fig. 2a), using data from Yala et al [10], RSS 1 (<2% cancer risk within 5 years), 2 (2 to <4%), and 3 (≥4%) patients were randomly assigned to 44% (eg, mean ± SD: 11 ± 3.1), 28% (7.1 ± 2.6), and 28% (7.1 ± 2.6), respectively, of the screening appointments on a given day (for calculations, see e-only Supplemental Methods). In contrast, the RSS template (Fig. 2b) fixed the number of RSS 3 and RSS 2 patients per day at seven to eight (7.1 ± 0.3; for calculations, see e-only Supplemental Methods) for each due to their higher cancer risk within 5 years than RSS 1 patients. Screening mammograms were structured so that all RSS 3 patients underwent examinations first (early in the day), then all RSS 2 patients, and finally all RSS 1 patients later in the day to reduce the likelihood of a patient late in the day requiring same-day diagnostic care. Although RSS categories were assigned in the non-RSS scenarios, they were not used to guide scheduling.

We assumed that patients with a higher risk of developing breast cancer within 5 years of their last screening mammogram would have a greater recall rate than those with a lower risk in the same time frame and that RSS 3 had a higher recall rate than RSS 1 and 2. We considered two hypothetical RSS recall rate distributions with uniform (RSS 1: 0.06, RSS 2: 0.12, RSS 3: 0.18) and nonuniform (RSS 1: 0.01, RSS 2: 0.01, RSS 3: 0.37) increases across three RSS categories (Table 1). The uniformly increasing recall rate distribution reflected the growing 5-year breast cancer risk across the three RSS categories (RSS 1: <2%, RSS 2: 2 to <4%, and RSS 3: ≥4%). The nonuniform recall rate distribution, which had the majority of recalls from RSS 3, indicated that controlling the number of RSS 3 cases would manage most of the recall variability. In the uniform scenario, RSS 1, 2, and 3 contributed 24%, 30%, and 46% of recalls, respectively (for calculations, see e-only Supplemental Methods), fixing the numbers of RSS 2 and 3 controls for 76% of recalls. In nonuniform scenarios, RSS 1, 2, and 3 contributed 4%, 2.5%, and 93.5% of recalls (for calculations, see e-only Supplemental Methods), with fixing the numbers of RSS 2 and 3 accounting for 96% of recalls. The overall recall rate remained at 11% in both RSS recall rate distributions, consistent with the average recall rate at our institution.

**Fig. 3.**
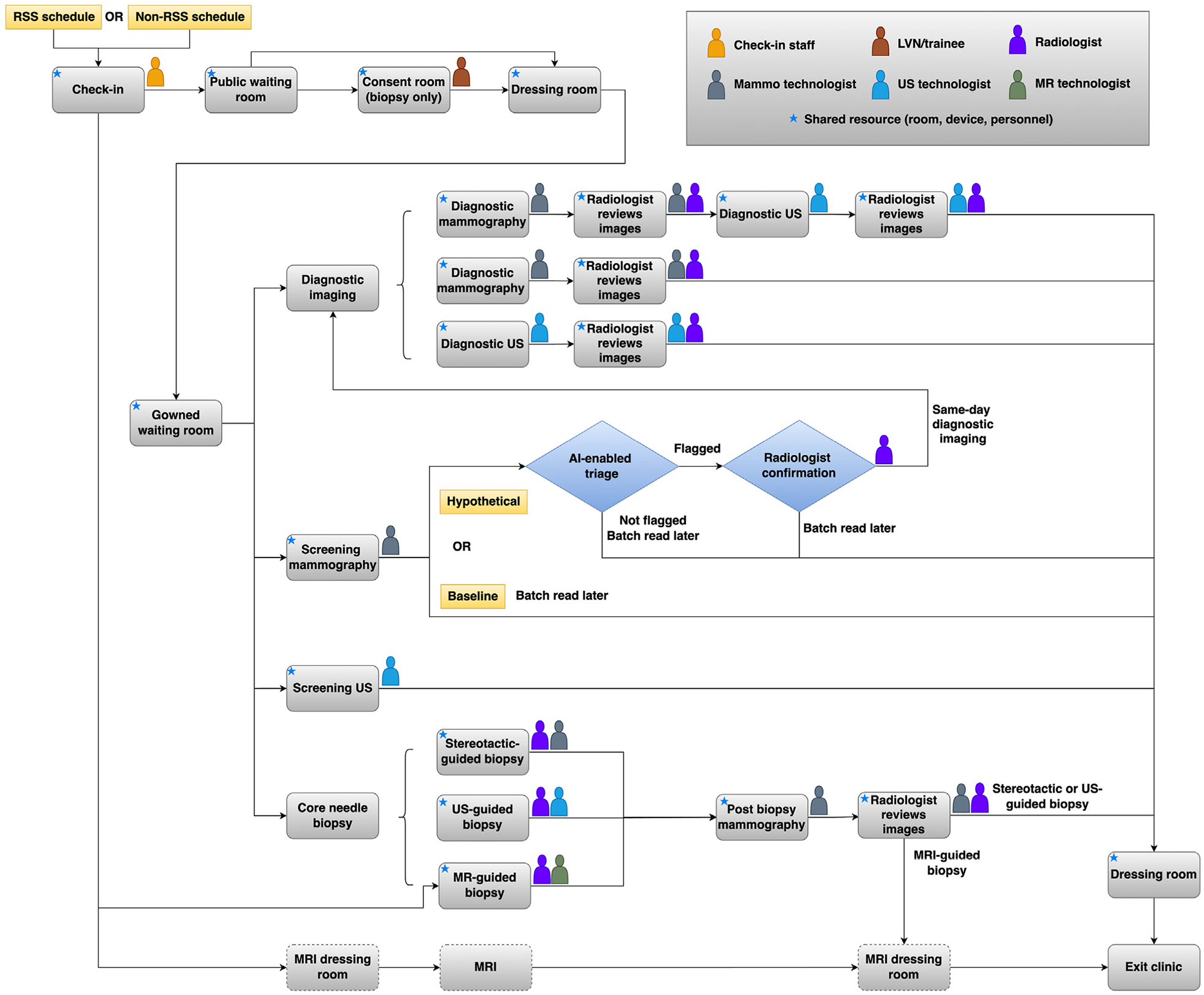
The patient workflows at the breast imaging center. Note: This figure was reproduced from Lin et al [7] with permission. AI = artificial intelligence; LVN = licensed vocational nurse; RSS = risk-stratified screening; US = ultrasound.

Figure 3 illustrates the baseline and hypothetical patient workflows featuring an AI-triaged same-day diagnostic imaging module. In the baseline workflow, screening mammograms are batch-read offline, with BI-RADS 0 (ie, a potentially abnormal result that is incomplete and necessitates further diagnostic evaluation) patients recalled for diagnostic workup at a later date. In the hypothetical AI-driven workflow, an immediate postscreening AI assessment generates a cancer risk score from 1 to 10 (ie, Transpara [ScreenPoint Medical], see e-only Supplemental Methods) [12]. Scores 1 to 7 would be reviewed as a batch, similar to the baseline workflow. Scores 8 to 10 confirmed as suspicious by a radiologist would qualify for same-day diagnostic imaging.

Model parameters, as shown in Table 1, were derived from 1 year of breast imaging scheduling data (December 1, 2022, to November 30, 2023), 2 years of breast imaging examination time stamps (January 1, 2022, to December 31, 2023), and 1 year of productivity data (June 1, 2022, to May 31, 2023). We used scheduling data to estimate the number of patients per hour (e-only Table S1 in the Supplement) and the distribution of patient types by hour (e-only Table S2 in the Supplement). We estimated a 10% no-show rate and an average of 20 days between a screening mammogram and a diagnostic examination.

### Model Assumptions

Our previous simulation studies have described most discrete event simulation model assumptions [6,7]. For instance, the model assumed all patients recommended for same-day diagnostic workups would complete the examinations and have insurance approval. Additionally, we assumed the days between a screening mammogram and a diagnostic workup followed a normal distribution (Table 1). In the hypothetical workflow, the AI algorithm ran throughout the day, and all radiologists and trainees could participate in caring for all types of patients.

### Outcomes and Analysis

The primary outcome of this study was the recall variance, defined as the variance in the number of daily recalls. In addition, we counted the number of weekdays with greater than twice the average number of daily recalls, as a higher-than-usual number of recall instances might overwhelm radiologists and technologists. Secondary outcomes included the average number of patients served within 10 operating hours, the average operating hours required to serve all checked-in patients, and the average patient waiting time across all patients. Among the eight scenarios (RSS versus non-RSS with or without same-day diagnostic workup using uniform versus nonuniform increasing recall rates), comparisons were made between the RSS versus non-RSS templates holding other parameters (ie, recall rate distribution and same-day diagnostic workup) constant. A sensitivity analysis was performed to examine possible outcomes for two scenarios: (1) a center offering only screening mammograms and same-day diagnostics, and (2) a center with the same setup as the primary analysis but with a 15% recall rate instead of 11% (see e-only Supplemental Methods).

### Statistical Analysis

We simulated 365 weekdays for each scenario and repeated with 10 different random seeds (see e-only Supplemental Methods). Results were averaged across the 10 iterations (ie, 365 × 10 = 3,650 weekdays). Comparisons were made between RSS and non-RSS simulation scenarios. Levene’s test compared the equality of two variances in the average number of recalled patients per day between the two scenarios. Wilcoxon signed-rank tests compared paired group means (ie, “per day” values including the average number of patients served within 10 operating hours and the average operating hours required to serve all checked-in patients), and Wilcoxon’s rank-sum tests compared unpaired group means (ie, “per patient” values including the average patient waiting time across all patients). A two-sided *P* value less than .05 was considered significant. Analysis was conducted using Python version 3.7.3 (Python Software Foundation, Wilmington, Delaware, United States) with the “SimPy” package, consistent with previous descriptions of simulation model components [6].

## RESULTS

### Model Verification and Validation

The simulation models had an average of 60 patients per day, the proportion of screening mammography patients was 42%, and the recall rate was 11% (approximately three recalls per day). These numbers aligned with the 1-year scheduling data from the imaging center. All scenarios adhered to the model parameters for the proportion of RSS categories and the overall and RSS-specific recall rates (see e-only Table S3 in the Supplement).

### Patient Recalls

Table 2 summarizes the patient recall results. When simulating using a uniformly increasing recall rate distribution, the recall variances were lower with RSS scheduling compared with non-RSS scheduling without same-day diagnostic workup (2.42 versus 2.77, *P* < .05). However, the reduction in recall variances between the RSS and non-RSS scheduling for scenarios with same-day diagnostic workup did not reach statistical significance (2.47 versus 2.58, *P* > .05). The RSS template resulted in a 30% reduction (57 versus 82) in the number of days with greater than twice the average number of recalls per day when no same-day diagnostic workup was implemented and a 28% reduction (54 versus 75) when same-day diagnostic workup was included.

Significant decreases in recall variances were noted when the simulation was executed using a nonuniformly increasing recall rate distribution. Transitioning from the non-RSS to the RSS template led to a 26% decrease in recall variance without same-day diagnostic workup (1.84 versus 2.48, *P* < .05) and a 30% decrease with same-day diagnostic workup (1.98 versus 2.82, *P* < .05). When comparing the RSS to non-RSS scheduling, the number of days with greater than twice the average number of recalls was reduced by 80% (12 versus 59) without same-day diagnostic workup and reduced by 76% (24 versus 98) with same-day diagnostic workup.

### Clinic Operation and Patient Waiting Time

The RSS template primarily impacted scenarios involving same-day diagnostic workups. Comparing RSS with non-RSS scheduling, there was a 0.7% (55.2 versus 54.8) and 1.3% (55.4 versus 54.7) increase in the average number of patients served within 10 hours per day for uniformly and nonuniformly increasing recall rate distribution scenarios (*P* < .05). Additionally, there was a 6-min (1.0%) and 12-min (2.0%) reduction in the average operating hours of the imaging center to serve all checked-in patients for the uniform (10.4 versus 10.5 hours, *P* < .05) and nonuniform (10.3 versus 10.5 hours, *P* < .05) scenario, respectively. However, transitioning to RSS scheduling resulted in a 0.6-min increase (0.21 versus 0.20 hours, *P* < .05) in average waiting time in the imaging center for uniform scenarios and a 2.4-min increase (0.24 versus 0.20 hours, *P* < .05) for nonuniform scenarios. Table 3 presents detailed comparisons of the scheduling approaches.

### Sensitivity Analyses

The first sensitivity analysis is a hypothetical clinic dedicated to screening mammograms and diagnostic imaging workups with or without AI-driven immediate interpretations of screening mammograms and same-day diagnostic workups (e-only Table S4 in the Supplement). The average number of recalls in this standalone clinic was six per day. The RSS schedule achieved outcomes similar to those observed in the current setting; however, the increased patient waiting time in the same-day diagnostic workup scenario was shorter compared with the current setting (eg, nonuniform, stand-alone versus current setting: 1.2 versus 2.4 min).

A second sensitivity analysis increased the recall rate to 15% (e-only Table S5 in the Supplement). The findings mirrored those of the 11% recall rate scenarios but with more pronounced effects. Implementing the RSS schedule resulted in a 10% to 49% reduction in recall variance across all scenarios (*P* < .05). Additionally, there was a 0.9% to 1.9% increase in the average number of patients served within 10 hours per day for scenarios involving same-day diagnostic imaging workups (*P* < .05) and a 0.9% (6 min) to 2.8% (18 min) decrease in average operating hours required to serve all checked-in patients in these scenarios (*P* < .05). However, compared with non-RSS scheduling, the average patient waiting times increased by 1.2 to 3 min (*P* < .05) across the same-day diagnostic imaging workup scenarios.

## DISCUSSION

Our simulations demonstrate that risk-based screening mammography scheduling could reduce recall variance and decrease the frequency of days with more than twice the average number of recalls, which might be challenging for a care team to manage. The RSS schedule achieves this by fixing the number of RSS 3 patients (the source of most recalls) and RSS 2 patients in a day. Moreover, caring for RSS 3 patients early in the day reduces the likelihood of a patient screened late in the day requiring a same-day diagnostic, which might require extending operational hours. Fewer days of higher postscreening diagnostics mean more days in which the current volume of patients could be cared for within 10 hours of imaging center operations when performing same-day diagnostic workups.

Notably, there are workflow considerations for any scheduling change. For example, concentrating on RSS 3 patients in the morning may lead to a busier clinic during that time, contributing to increased waiting times after transitioning to the RSS template. To mitigate these effects, alternative RSS templates could be considered, such as (1) prioritizing both RSS 2 and RSS 3 patients at the beginning of the day (Fig. 1a in the e-only Supplement) or (2) prioritizing all RSS 2 and RSS 3 patients along with a small portion of RSS 1 patients early in the day (Fig. 1b in the e-only Supplement). The goal is to reorganize the sequence of RSS categories to avoid overwhelming the radiologists and technologists while minimizing extended patient wait times by carefully controlling the daily numbers of RSS 2 and 3 patients to manage recall variance effectively.

The screening mammography patient volume in this study is relatively low, averaging 25 patients per day, due to the distribution of examinations across 12 sites within our institution. A sensitivity analysis of a hypothetical stand-alone screening clinic suggests that imaging centers with higher volumes (eg, 55 examinations per day) of screening mammography patients could benefit from implementing the RSS schedule for same-day diagnostic workups. Besides, the effectiveness of the RSS schedule depends on the recall rate and reducing daily fluctuations in the number of RSS categories responsible for the majority of recalls. The national screening mammography performance measures show a mean recall rate of 10% to 12% [13,14], yet previous studies have reported recall rates exceeding 12% [15–18]. As the recall rate increases, so does recall variance. Under a 15% recall rate, the impact of the RSS schedule becomes more pronounced. Moreover, the nonuniform scenario demonstrated more considerable effects across simulations than the uniform scenario. In the uniform scenario, RSS 2 and 3 contributed 76% of recalls. In nonuniform scenarios, they accounted for 96% of the recalls. This suggests that although actual recall rates for each RSS category may vary, fixing the number of RSS categories contributing to as many recalls as possible would maximize the effectiveness of the RSS schedule. Successful control of recall variance could minimize the risk of overwhelming same-day diagnostic workups and potentially biopsy volumes on any given day, enabling their more effective implementation to mitigate delays in diagnosis and improve health equity [19,20].

### Limitations

The limitations of the discrete event simulation models have been discussed in our previous work [6,7]. This study estimated variation using averages in the simulation model. However, alternative methods for analyzing variation might be more intuitive to clinicians and better capture the day-to-day fluctuations they encounter (eg, a simulation showing possible outcomes across 365 days to visualize what care teams might experience). This study did not consider a range of workflow options that could affect the study outcomes and may be pragmatic solutions to extreme days, such as enabling a center to stop diagnostics at any point during the day. Additionally, risk-based scheduling reduces appointment options for patients, as RSS 1 patients are restricted to afternoon appointments and RSS 3 patients to mornings, potentially leading to access issues. Alternative RSS scheduling templates (see Fig. S1 in the e-only Supplement) could offer greater flexibility. The RSS category distribution was derived from a published study rather than our institution’s data. In the future, we may consider using the state-of-the-art deep learning model to verify the RSS distribution using data from our institution. We hypothesized that RSS 3 would have the highest recall rate due to its association with the highest cancer risk within 5 years, but this assumption requires validation with real-world data. The agreement between RSS-based recalls and those confirmed by AI and radiologists in same-day diagnostic workups needs to be evaluated during the pilot implementation. Patient recalls after potentially abnormal screening mammograms involve multiple factors, and simulations capture only specific drivers of recall variations, such as varying recall rates. Future research could further investigate RSS categories using actual patient data, mainly focusing on patient age and breast density, major contributors to recalls. AI could aid in the early recognition of benign cases, potentially reducing false positive diagnostic imaging and enhancing diagnostic accuracy and efficiency.

## Supplementary Material

1

## Figures and Tables

**Table 1.** Parameters of discrete event simulation models

Name	Personnel	Capacity	Condition	Distribution
Operating hours	NA	NA	Stops patient check-in at 9.5 h and closes at 10 h	NA
Patient arrival	NA	NA	Arrival rate: varying hourly	Poisson process
			Mean interarrival time: 1/arrival rate	
Patient check-in	Staff	3	NA	Normal (0.05 h, 0.01 h)
Public waiting room	NA	20	NA	Normal (0.17 h, 0.034 h)
Consent room	LVN/trainee	1	NA	Normal (0.17 h, 0.034 h)
Dressing room	NA	3	NA	Normal (0.03 h, 0.006 h)
Gowned waiting room	NA	5	NA	Normal (0.017 h, 0.0034 h)
AI assessment (hypothetical workflow only)	Radiologist/trainee	4	100% agreement with RSS recalls	Normal (0.25 h, 0.05 h)
Screening mammography	Mammo technologist	3 mammo	Varying hourly	Normal (0.17 h, 0.034 h)
			RSS 1: 44%; RSS 2: 28%; RSS 3: 28% Uniform: RSS 1 recall: 6%; RSS 2 recall: 12%; RSS 3 recall: 18% Nonuniform: RSS 1 recall: 1%; RSS 2 recall: 1%; RSS 3 recall: 37%	Recall diagnostic imaging in how many days: normal (20 d, 4 d) Recall diagnostic imaging slot number: random (1, average daily patient volume over a year × 0.67)
Screening US	US technologist	2 US	Varying hourly	Normal (0.25 h, 0.05 h)
Diagnostic imaging	Mammo/US technologist	3 mammo, 2 US	Subtypes: diagnostic mammo + diagnostic US, diagnostic mammo, and diagnostic US Varying hourly, except for same-day diagnostic imaging after a screening mammogram: 70% diagnostic mammo + diagnostic US, 15% diagnostic mammo, and 15% diagnostic US	Diagnostic mammo: normal (0.417 h, 0.0834 h); diagnostic US: normal (0.417 h, 0.0834 h)
Diagnostic imaging review	Radiologist/trainee	4	NA	Normal (0.083 h, 0.017 h)
Core needle biopsy	Mammo/US/MR technologist, radiologist, LVN/trainee	3 mammo, 2 US, 4 radiologists/trainees	Subtypes: stereotactic-guided, US-guided, and MR-guided	US-guided: normal (0.75 h, 0.15 h); Stereotactic-guided: normal (0.75 h, 0.15 h); MR-guided: normal (0.5 h, 0.1 h)
			Varying hourly	
MRI	MR technologist	NA	NA	NA
No-show	NA	NA	NA	10%

In the Distribution column, “normal (X, Y)” represents a normal distribution with a mean of X and an SD of Y. Imaging equipment is shared resources across all imaging examinations and procedures (eg, mammography equipment can be used for screening mammography, diagnostic mammography, or stereotactic-guided biopsy). Radiologists are allocated across all tasks that require their expertise. MRI examinations are deemed irrelevant within the workflows because they do not share resources with other examinations or procedures. AI = artificial intelligence; LVN = licensed vocational nurse; mammo = mammogram; NA = not applicable; RSS = risk-stratified screening; US = ultrasound.

**Table 2.** Simulation results on recalls

Recall Rate Distribution	Schedule	Same-Day Diagnostic Workup	Average Number of Recalls/Day (SD)	Variance: Recall/Day	*P* Value*	Number of Days With > 6 Recalls (%)
Uniform	No RSS	No	2.8 (1.66)	2.77	NA	82 (2.2)
RSS 1: 0.06	RSS	No	2.8 (1.55)	2.42	<.001	57 (1.6)
RSS 2: 0.12	No RSS	Yes	2.7 (1.61)	2.58	NA	75 (2.1)
RSS 3: 0.18	RSS	Yes	2.7 (1.57)	2.47	0.39	54 (1.5)
Nonuniform	No RSS	No	2.8 (1.58)	2.48	NA	59 (1.6)
RSS 1: 0.01	RSS	No	2.7 (1.36)	1.84	<.001	12 (0.3)
RSS 2: 0.01	No RSS	Yes	2.9 (1.68)	2.82	NA	98 (2.7)
RSS 3: 0.37	RSS	Yes	2.8 (1.41)	1.98	<.001	24 (0.7)

* A Levene’s test *P* value was obtained by comparing the variances between the scenarios with and without RSS. AI = artificial intelligence; NA = not applicable; RSS = risk-stratified screening.

**Table 3.** Simulation results on clinical operation and patient waiting time

Recall Rate Distribution	Schedule	Same-Day Diagnostic Workup	Average Number of Patients/Day Served Within 10 h (SD)	*P* Value*	Average operating Hours/Day to Serve All Checked-In Patients (SD)	*P* Value*	Average Waiting Time Among Patients Served Within 10 h (SD)	*P* Value^†^
Uniform	Non-RSS	No	59.2 (3.4)	NA	10.2 (0.4)	NA	0.19 (0.32)	NA
RSS 1: 0.06	RSS	No	59.1 (3.2)	.16	10.2 (0.4)	.017	0.19 (0.33)	.38
RSS 2: 0.12	Non-RSS	Yes	54.8 (3.7)	NA	10.5 (0.5)	NA	0.20 (0.34)	NA
RSS 3: 0.18	RSS	Yes	55.2 (3.9)	<.001	10.4 (0.5)	<.001	0.21 (0.35)	<.001
Nonuniform	Non-RSS	No	59.2 (3.3)	NA	10.2 (0.4)	NA	0.20 (0.34)	NA
RSS 1: 0.01	RSS	No	59.1 (3.5)	.07	10.2 (0.4)	.16	0.19 (0.33)	<.001
RSS 2: 0.01	Non-RSS	Yes	54.7 (3.7)	NA	10.5 (0.5)	NA	0.20 (0.34)	NA
RSS 3: 0.37	RSS	Yes	55.4 (3.6)	<.001	10.3 (0.4)	<.001	0.24 (0.39)	<.001

AI = artificial intelligence; NA = not applicable; RSS = risk-stratified screening.

* A Wilcoxon signed-rank test *P* value was derived by comparing the means from the RSS and non-RSS scenarios.

† A Wilcoxon rank-sum test *P* value was derived by comparing the means from the RSS and non-RSS scenarios.

## TAKE-HOME POINTS

- An RSS scheduling mechanism could reduce recall variance, the variation in the daily number of recalled screening mammograms, compared with a conventional nonrisk-stratified schedule.
- When incorporating same-day diagnostic services, RSS scheduling may allow clinics to better accommodate planned (scheduled) and unplanned (same-day) diagnostic care within normal operational hours without the need for extended hours.
- RSS templates may be valuable in screening centers that adopt same-day diagnostics.
