# Supplementary material for: Risk-Stratified Screening: A Simulation Study of Scheduling Templates on Daily Mammography Recalls
Source: J Am Coll Radiol. Author manuscript; Available in PMC 2025 Mar 20. (PMC11925393; doi:10.1016/j.jacr.2024.12.010)
Supplement: 1 [file NIHMS2064821-supplement-1.docx]

**Supplemental Materials for “Risk-Stratified Screening (RSS): A Simulation Study of Scheduling Templates on Daily Mammography Recall Rates”**

**Supplemental Methods**

**Figure S1**. Examples of alternative RSS scheduling templates.

**Table S1**. Simulation model parameters: average number of patients per hour.

**Table S2**. Simulation model parameters: percentages of examinations by hour.

**Table S3**. Validation of simulation models.

**Table S4.** Sensitivity analyses for the standalone screening clinic scenario.

**Table S5.** Sensitivity analyses under a 15% recall rate.

**References**

**Supplemental Methods**

**Derivation of the RSS categories using a hybrid deep learning model:** The study by Yala et al.^1^ reviewed 88,994 mammograms from 39,571 women between 2009 and 2012, dividing them into 71,689 for training, 8,554 for validation, and 8,751 for testing, aiming to develop a mammography-based deep-learning breast cancer risk model. Combining image information and risk factors (available to the Tyrer-Cuzick model version 8 and race and prior history of breast cancer), the hybrid deep learning model achieved the highest area under the receiver operating characteristics curve on the test set (AUC=0.70). This model was developed to predict breast cancer risk within 5 years and not for clinical workflow solutions.

**Calculation of RSS proportions**: Based on **Figure 5a** in Yala et al.^1^, we choose the following risk thresholds RSS 1 (<2% cancer risk within five years), 2 (2 to <4% cancer risk within five years), and 3 (≥4% cancer risk within five years) in order to assign the largest percentage of the screening population to the lowest risk-stratified screening group (RSS 1) and fewer to RSS 2 and 3 where most recall variations occur. There were 3818 subjects with <2% risk (risk-stratified screening [RSS] 1), 2449 with 2% to <4% risk (RSS 2), and 2484 with ≥4% risk (RSS 3). The total number of subjects was 8751. Therefore, the proportions of RSS 1, 2, and 3 were 44% (3818/8751), 28% (2449/8751), and 28% (2484/8751), respectively.

**Creation of the RSS template**: Initially, we simulated the scenario without RSS, the scheduling template, collecting data on screening mammography patients, their assigned RSS categories, and recall information. Subsequently, we simulated the RSS scenario, randomly selecting one patient from the pool of screening mammography patients without replacement. This allowed us to rearrange the same pool of screening mammography patients by their RSS categories in the RSS scenarios, ensuring consistency when comparing the effects of different scheduling templates.

**Determination of the number of RSS 2 or 3 exams in the RSS template**: Out of 60 patients per day, 42% (or approximately 25.2) are screening mammography patients. Given that 28% of these screening patients were categorized as RSS 2 and 28% as RSS 3, we expected, on average, about 7.1 RSS 2 and 7.1 RSS 3 patients per day. Therefore, the expected number of RSS 2 and RSS 3 patients was set to be between seven to eight daily.

**AI model used to triage screening mammography patients**: Transpara (ScreenPoint Medical) is a deep learning-based system designed to automate breast cancer detection in mammography and breast tomosynthesis processed images. It generates a score between 1 and 10, representing the likelihood of detecting visible cancer in an exam, with 10 indicating the highest probability. Previous studies have investigated Transpara^2-8^, which we used to define the AI model performance characteristics (e.g., true positive rate, false positive rate) in our simulation.

**Warm-up period in the simulations**: In this study, Day 1 of the simulation represented the start of a new breast imaging center, with no diagnostic mammography patients following screening mammograms. From Day 1, the baseline workflow allowed patients who required diagnostic workups after a BI-RADS 0 screening mammogram to begin scheduling follow-up exams ("recalls"). On average, there was a 20-day gap between the screening and the follow-up diagnostic workup. Consequently, around Day 20, recalled patients would start arriving for their follow-up exams. By Day 30, the simulation system reached stability, and data collection for analysis commenced.

**Sensitivity analyses**: The first converted the target breast imaging center into a standalone screening clinic, focusing exclusively on screening mammograms and diagnostic imaging workups following abnormal screening results. The clinic maintained the same resources as the target site in the primary analysis, with an average of seven patients per hour over 9.5 hours for check-in, aligning with the target site's daily average of 60 patients. The second sensitivity analysis increased the overall recall rate to 15% from 11%. Consequently, the recall rate distributions in each RSS category adjusted for both uniform (RSS 1: 0.08, RSS 2: 0.16, RSS 3: 0.25) and non-uniform (RSS 1: 0.01, RSS 2: 0.01, RSS 3: 0.51) scenarios.

**Proportions of recalls from each RSS category**

| RSS proportions  RSS 1: 0.44  RSS 2: 0.28  RSS 3: 0.28 | Uniform RSS recall rate  RSS 1: 0.06  RSS 2: 0.12  RSS 3: 0.18 | Non-uniform RSS recall rate  RSS 1: 0.01  RSS 2: 0.01  RSS 3: 0.37 |
| --- | --- | --- |
| Proportion of RSS 1 among all recalls | 0.44*0.06/(0.44*0.06+0.28*0.12+0.28*0.18)=0.24 | 0.44*0.01/(0.44*0.01+0.28*0.01+0.28*0.37)=0.040 |
| Proportion of RSS 2 among all recalls | 0.28*0.12/(0.44*0.06+0.28*0.12+0.28*0.18)=0.30 | 0.28*0.01/(0.44*0.01+0.28*0.01+0.28*0.37)=0.025 |
| Proportion of RSS 3 among all recalls | 0.28*0.18/(0.44*0.06+0.28*0.12+0.28*0.18)=0.46 | 0.28*0.37/(0.44*0.01+0.28*0.01+0.28*0.37)=0.935 |


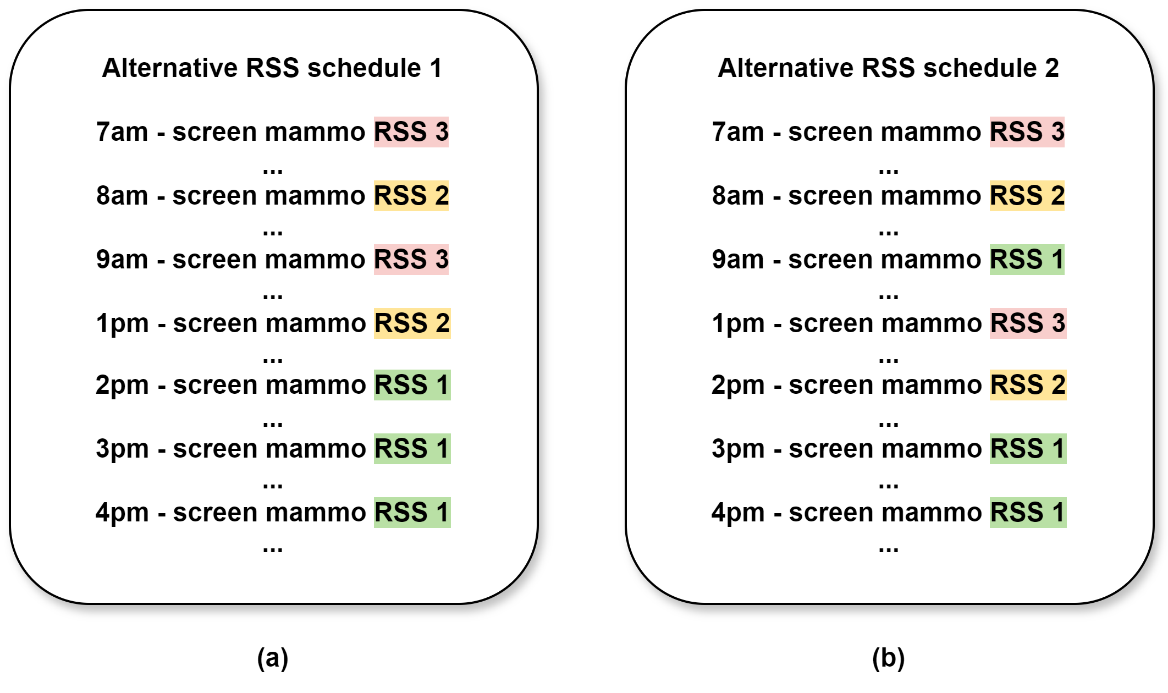


**Figure S1**. Examples of alternative RSS scheduling templates. (a) Prioritizing both RSS 2 and RSS 3 patients at the beginning of the day and (b) prioritizing all RSS 2 and RSS 3 patients along with a small portion of RSS 1 patients early in the day. The patient may only book an appointment that matches their risk of developing breast cancer. **Abbreviations**: RSS: risk-stratified screening.

| **Table S1**. Simulation model parameters: average number of patients per hour. | |
| --- | --- |
| Hour | Average number of patients |
| 7 am to 8 am | 5.87 |
| 8 am to 9 am | 8.30 |
| 9 am to 10 am | 8.11 |
| 10 am to 11 am | 7.50 |
| 11 am to 12 pm | 7.09 |
| 12 pm to 1 pm | 2.38 |
| 1 pm to 2 pm | 8.83 |
| 2 pm to 3 pm | 7.51 |
| 3 pm to 4 pm | 8.59 |
| 4 pm to 5 pm | 2.91 |

| **Table S2**. Simulation model parameters: percentages of examinations by hour. | | | | | | | | | | |
| --- | --- | --- | --- | --- | --- | --- | --- | --- | --- | --- |
| Exam type | 7 am to 8 am | 8 am to 9 am | 9 am to 10 am | 10 am to 11 am | 11 am to 12 pm | 12 pm to 1 pm | 1 pm to 2 pm | 2 pm to 3 pm | 3 pm to 4 pm | 4 pm to 5 pm |
| Stereotactic-guided biopsy | 0.00 | 0.00 | 0.00 | 0.00 | 0.00 | 0.00 | 0.00 | 0.00 | 0.00 | 0.00 |
| MR-guided biopsy | 0.00 | 0.00 | 0.00 | 0.00 | 0.00 | 0.00 | 0.05 | 0.00 | 0.01 | 0.00 |
| US-guided biopsy | 0.00 | 0.00 | 0.08 | 0.08 | 0.03 | 0.00 | 0.03 | 0.04 | 0.00 | 0.00 |
| Diagnostic mammo | 0.01 | 0.07 | 0.02 | 0.03 | 0.07 | 0.41 | 0.03 | 0.03 | 0.01 | 0.01 |
| Diagnostic mammo + diagnostic US | 0.20 | 0.46 | 0.26 | 0.33 | 0.08 | 0.36 | 0.28 | 0.34 | 0.15 | 0.02 |
| Diagnostic US | 0.02 | 0.18 | 0.09 | 0.08 | 0.02 | 0.04 | 0.15 | 0.06 | 0.02 | 0.01 |
| MRI | 0.12 | 0.05 | 0.09 | 0.10 | 0.05 | 0.02 | 0.03 | 0.03 | 0.03 | 0.07 |
| Screen mammo | 0.65 | 0.20 | 0.41 | 0.34 | 0.51 | 0.17 | 0.40 | 0.47 | 0.42 | 0.86 |
| Screen US | 0.00 | 0.04 | 0.05 | 0.04 | 0.24 | 0.01 | 0.02 | 0.03 | 0.34 | 0.02 |
| **Abbreviations**: US: ultrasound; MR: magnetic resonance; MR: magnetic resonance; MRI: magnetic resonance imaging. | | | | | | | | | | |

| **Table S3**. Validation of simulation models. | | | | | | | | | | | |
| --- | --- | --- | --- | --- | --- | --- | --- | --- | --- | --- | --- |
| Recall rate distribution | Schedule | Same-day diagnostic workup | Average number of screening mammo patients/day (sd) | | | |  | Recall rate | | | |
|  |  |  | Total | RSS 1 | RSS 2 | RSS 3 |  | RSS 1 | RSS 2 | RSS 3 | Overall |
| Gradient RSS 1: 0.06 RSS 2: 0.12 RSS 3: 0.18 | No RSS | No | 25.3 (4.1) | 11.0 (3.1) | 7.1 (2.5) | 7.1 (2.5) |  | 0.06 | 0.13 | 0.18 | 0.11 |
|  | RSS | No | 25.1 (4.1) | 10.9 (4.1) | 7.1 (0.3) | 7.1 (0.2) |  | 0.06 | 0.12 | 0.18 | 0.11 |
|  | No RSS | Yes | 25.1 (4.2) | 11.1 (3.2) | 7.0 (2.5) | 7.1 (2.6) |  | 0.06 | 0.11 | 0.18 | 0.11 |
|  | RSS | Yes | 25.1 (4.2) | 11.0 (4.2) | 7.1 (0.4) | 7.1 (0.3) |  | 0.06 | 0.11 | 0.18 | 0.11 |
| Unbalanced RSS 1: 0.01 RSS 2: 0.01 RSS 3: 0.37 | No RSS | No | 25.1 (4.2) | 11.0 (3.1) | 7.1 (2.6) | 7.0 (2.5) |  | 0.01 | 0.01 | 0.37 | 0.11 |
|  | RSS | No | 25.1 (4.1) | 11.0 (4.1) | 7.0 (0.3) | 7.1 (0.2) |  | 0.01 | 0.01 | 0.36 | 0.11 |
|  | No RSS | Yes | 25.0 (4.2) | 11.0 (3.1) | 7.0 (2.5) | 7.1 (2.5) |  | 0.01 | 0.01 | 0.37 | 0.11 |
|  | RSS | Yes | 24.9 (4.1) | 10.7 (4.2) | 7.1 (0.3) | 7.1 (0.3) |  | 0.01 | 0.01 | 0.38 | 0.11 |
| Abbreviations: mammo: mammography; AI: artificial intelligence; sd: standard deviation; RSS: risk stratifying screening. | | | | | | | | | | | |

| **Table S4.** Sensitivity analyses for the standalone screening clinic scenario. | | | | | | | | | | | | | | | | | | | | |
| --- | --- | --- | --- | --- | --- | --- | --- | --- | --- | --- | --- | --- | --- | --- | --- | --- | --- | --- | --- | --- |
| Recall rate distribution | Schedule | Same-day diagnostic workup | Average number of screening mammo patients/day (sd) | | | | | Variance: recall/day | p-value ^a^ |  | Recall rate | | |  | Average number of patients/day served within 10 hours (sd) | p-value ^b^ | Average operating hours/day to serve all checked-in patients (sd) | p-value ^b^ | Average waiting time among patients served within 10 hours (sd) | p-value ^c^ |
|  |  |  | Total | RSS 1 | RSS 2 | RSS 3 | Recall |  |  |  | RSS 1 | RSS 2 | RSS 3 |  |  |  |  |  |  |  |
| Uniform | Non-RSS | No | 54.8 (7.8) | 24.1 (5.0) | 15.4 (4.0) | 15.4 (4.0) | 6.1 (2.5) | 6.31 | NA |  | 0.06 | 0.12 | 0.18 |  | 59.9 (7.6) | NA | 9.8 (0.2) | NA | 0.01 (0.07) | NA |
| RSS 1: 0.06 | RSS | No | 54.6 (7.8) | 23.6 (7.9) | 15.5 (0.5) | 15.5 (0.5) | 6.1 (2.3) | 5.47 | <0.001 |  | 0.06 | 0.12 | 0.18 |  | 59.7 (7.7) | 0.002 | 9.8 (0.2) | 0.84 | 0.01 (0.07) | 0.86 |
| RSS 2: 0.12 | Non-RSS | Yes | 54.5 (7.9) | 24.1 (5.1) | 15.2 (4.0) | 15.2 (3.9) | 6.0 (2.5) | 6.22 | NA |  | 0.06 | 0.12 | 0.18 |  | 50.8 (6.9) | NA | 10.6 (0.5) | NA | 0.17 (0.26) | NA |
| RSS 3: 0.18 | RSS | Yes | 54.4 (8.0) | 23.8 (8.0) | 15.3 (0.5) | 15.3 (0.5) | 6.0 (2.3) | 5.29 | 0.002 |  | 0.06 | 0.12 | 0.18 |  | 51.1 (7.0) | 0.07 | 10.5 (0.5) | <0.001 | 0.17 (0.26) | <0.001 |
| Non-uniform | Non-RSS | No | 54.7 (8.0) | 24.2 (5.1) | 15.2 (4.0) | 15.2 (4.1) | 6.1 (2.5) | 6.24 | NA |  | 0.01 | 0.01 | 0.37 |  | 59.7 (7.8) | NA | 9.8 (0.2) | NA | 0.01 (0.07) | NA |
| RSS 1: 0.01 | RSS | No | 54.5 (7.7) | 23.8 (7.7) | 15.3 (0.5) | 15.3 (0.5) | 6.1 (2.0) | 3.95 | <0.001 |  | 0.01 | 0.01 | 0.37 |  | 59.5 (7.5) | 0.51 | 9.8 (0.2) | 0.04 | 0.01 (0.07) | 0.34 |
| RSS 2: 0.01 | Non-RSS | Yes | 54.4 (7.9) | 24.0 (5.1) | 15.2 (4.0) | 15.2 (4.0) | 6.1 (2.5) | 6.35 | NA |  | 0.01 | 0.01 | 0.38 |  | 50.6 (6.9) | NA | 10.7 (0.5) | NA | 0.17 (0.26) | NA |
| RSS 3: 0.37 | RSS | Yes | 54.1 (7.8) | 23.4 (7.8) | 15.3 (0.5) | 15.3 (0.5) | 6.1 (2.0) | 4.13 | <0.001 |  | 0.01 | 0.01 | 0.37 |  | 51.5 (7.0) | <0.001 | 10.3 (0.3) | <0.001 | 0.19 (0.27) | <0.001 |
| **Notes**: ^a^ A Levene's test p-value was obtained by comparing the variances between the scenarios with and without RSS. ^b^ A Wilcoxon signed rank test p-value was derived by comparing the means from the RSS and non-RSS scenarios. ^c^ A Wilcoxon rank sum test p-value was derived by comparing the means from the RSS and non-RSS scenarios. | | | | | | | | | | | | | | | | | | | | |
| **Abbreviations**: RSS: risk stratifying screening; AI: artificial intelligence; sd: standard deviation; RSS: risk stratifying screening. | | | | | | | | | | | | | | | | | | | | |

| **Table S5**. Sensitivity analyses under a 15% recall rate. | | | | | | | | | | | | | | | | | | | | |
| --- | --- | --- | --- | --- | --- | --- | --- | --- | --- | --- | --- | --- | --- | --- | --- | --- | --- | --- | --- | --- |
| Recall rate distribution | Schedule | Same-day diagnostic workup | Average number of screening mammo patients/day (sd) | | | | | Variance: recall/day | p-value ^a^ |  | Recall rate | | |  | Average number of patients/day served within 10 hours (sd) | p-value ^b^ | Average operating hours/day to serve all checked-in patients (sd) | p-value ^b^ | Average waiting time among patients served within 10 hours (sd) | p-value ^c^ |
|  |  |  | Total | RSS 1 | RSS 2 | RSS 3 | Recall |  |  |  | RSS 1 | RSS 2 | RSS 3 |  |  |  |  |  |  |  |
| Uniform RSS 1: 0.08 RSS 2: 0.16 RSS 3: 0.25 | Non-RSS | No | 25.0 (4.2) | 11.0 (3.1) | 7.0 (2.6) | 7.1 (2.6) | 3.8 (1.9) | 3.48 | NA |  | 0.08 | 0.16 | 0.25 |  | 59.2 (3.5) | NA | 10.2 (0.4) | NA | 0.20 (0.33) | NA |
|  | RSS | No | 24.8 (3.9) | 10.7 (3.9) | 7.0 (0.2) | 7.0 (0.2) | 3.7 (1.8) | 3.14 | 0.003 |  | 0.08 | 0.16 | 0.25 |  | 59.1 (3.3) | 0.009 | 10.2 (0.4) | 0.31 | 0.19 (0.33) | <0.001 |
|  | Non-RSS | Yes | 24.7 (4.3) | 10.9 (3.2) | 6.8 (2.5) | 6.9 (2.5) | 3.7 (1.9) | 3.55 | NA |  | 0.08 | 0.15 | 0.25 |  | 53.8 (4.0) | NA | 10.6 (0.5) | NA | 0.20 (0.34) | NA |
|  | RSS | Yes | 24.9 (4.1) | 10.9 (4.1) | 7.0 (0.2) | 7.0 (0.2) | 3.7 (1.8) | 3.12 | <0.001 |  | 0.08 | 0.15 | 0.25 |  | 54.3 (3.7) | <0.001 | 10.5 (0.5) | <0.001 | 0.22 (0.36) | <0.001 |
| Non-uniform RSS 1: 0.01 RSS 2: 0.01 RSS 3: 0.51 | Non-RSS | No | 24.8 (4.2) | 10.9 (3.1) | 6.9 (2.6) | 6.9 (2.5) | 3.7 (1.9) | 3.43 | NA |  | 0.009 | 0.01 | 0.50 |  | 59.1 (3.4) | NA | 10.2 (0.4) | NA | 0.20 (0.34) | NA |
|  | RSS | No | 24.8 (4.1) | 10.8 (4.1) | 7.0 (0.2) | 7.0 (0.1) | 3.7 (1.4) | 1.90 | <0.001 |  | 0.009 | 0.01 | 0.51 |  | 59.1 (3.2) | 0.64 | 10.2 (0.4) | 0.06 | 0.20 (0.34) | <0.001 |
|  | Non-RSS | Yes | 24.7 (4.2) | 10.9 (3.1) | 6.8 (2.5) | 6.9 (2.5) | 3.7 (1.9) | 3.71 | NA |  | 0.01 | 0.01 | 0.51 |  | 53.6 (4.1) | NA | 10.6 (0.5) | NA | 0.20 (0.34) | NA |
|  | RSS | Yes | 24.6 (4.2) | 10.6 (4.2) | 7.0 (0.4) | 7.0 (0.3) | 3.7 (1.4) | 1.91 | <0.001 |  | 0.009 | 0.01 | 0.51 |  | 54.6 (4.1) | <0.001 | 10.3 (0.4) | <0.001 | 0.25 (0.41) | <0.001 |
| **Notes**: ^a^ A Levene's test p-value was obtained by comparing the variances between the scenarios with and without RSS. ^b^ A Wilcoxon signed rank test p-value was derived by comparing the means from the RSS and non-RSS scenarios. ^c^ A Wilcoxon rank sum test p-value was derived by comparing the means from the RSS and non-RSS scenarios. | | | | | | | | | | | | | | | | | | | | |
| **Abbreviations**: mammo: mammography; RSS: risk stratifying screening; AI: artificial intelligence; SD: standard deviation; RSS: risk stratifying screening. | | | | | | | | | | | | | | | | | | | | |

**References**:

1. Yala A, Lehman C, Schuster T, Portnoi T, Barzilay R. A Deep Learning Mammography-based Model for Improved Breast Cancer Risk Prediction. *Radiology*. Jul 2019;292(1):60-66. doi:10.1148/radiol.2019182716

2. Rodriguez-Ruiz A, Lång K, Gubern-Merida A, et al. Can we reduce the workload of mammographic screening by automatic identification of normal exams with artificial intelligence? A feasibility study. *Eur Radiol*. Sep 2019;29(9):4825-4832. doi:10.1007/s00330-019-06186-9

3. Rodriguez-Ruiz A, Lång K, Gubern-Merida A, et al. Stand-Alone Artificial Intelligence for Breast Cancer Detection in Mammography: Comparison With 101 Radiologists. *Jnci-J Natl Cancer I*. Sep 2019;111(9):916-922. doi:ARTN djy222

10.1093/jnci/djy222

4. Christiana B, Rodriguez-Ruiz A, Mieskes C, Karssemeijer N, Heywang-Köbrunner SH. Going from double to single reading for screening exams labeled as likely normal by AI: what is the impact? *Proc Spie*. 2020;11513doi:Artn 115130d

10.1117/12.2564179

5. Rodriguez-Ruiz A, Krupinski E, Mordang JJ, et al. Detection of Breast Cancer with Mammography: Effect of an Artificial Intelligence Support System. *Radiology*. Feb 2019;290(2):305-314. doi:10.1148/radiol.2018181371

6. Sasaki M, Tozaki M, Rodríguez-Ruiz A, et al. Artificial intelligence for breast cancer detection in mammography: experience of use of the ScreenPoint Medical Transpara system in 310 Japanese women. *Breast Cancer-Tokyo*. Jul 2020;27(4):642-651. doi:10.1007/s12282-020-01061-8

7. Dustler M, Dahlblom V, Tingberg A, Zackrisson S. The effect of breast density on the performance of deep learning-based breast cancer detection methods for mammography. *Proc Spie*. 2020;11513doi:Artn 1151324

10.1117/12.2564328

8. Raya-Povedano JL, Romero-Martin S, Elias-Cabot E, Gubern-Merida A, Rodriguez-Ruiz A, Alvarez-Benito M. AI-based Strategies to Reduce Workload in Breast Cancer Screening with Mammography and Tomosynthesis: A Retrospective Evaluation. *Radiology*. Jul 2021;300(1):57-65. doi:10.1148/radiol.2021203555
